# A Cross-Sectional Study of the Prevalence and Determinants of Formula-Feeding Practices by Mothers in a Sub-district of Kerala, India

**DOI:** 10.7759/cureus.74706

**Published:** 2024-11-28

**Authors:** Latha Bhagyalekshmi, Chitra Tomy, Jeby J Olickal, Kavumpurathu R Thankappan

**Affiliations:** 1 Department of Public Health, Amrita Institute of Medical Sciences, Kochi, IND; 2 Department of Community Medicine, Amrita Institute of Medical Sciences, Kochi, IND

**Keywords:** exclusive breast feeding, feeding infants and young children, formula feeding, low birth weight, private hospital

## Abstract

Background

There is a scarcity of data on formula-feeding practices in India. Therefore, we conducted this study to determine the prevalence and factors associated with formula-feeding practices among mothers of infants in a sub-district of Kerala, India.

Methods

This community-based cross-sectional study included 300 mothers of infants aged 0-12 months selected using multistage cluster sampling. Data on formula feeding practices were collected at any point during the infant’s life through a pre-tested, structured interview schedule. Log-binomial regression analysis was conducted to identify factors associated with formula feeding.

Results

The mean age of the mothers was 27.06 years (SD: 3.90). Of the participants, 75% (n=225) were graduates or postgraduates, 67.33% (n=202) were homemakers, and 58% (n=174) were above the poverty line (APL). Early initiation of breastfeeding (within one hour of birth) was reported by 65% (n=195) of the mothers. The prevalence of formula feeding among infants under one year was 53.3% [95% Confidence Interval (CI): 47.5-59.1%]. Infants of mothers who delivered in private hospitals [adjusted prevalence ratio (APR): 3.44, 95% CI: 2.47-4.79; p<0.001] and those with a birth weight below 2.5 kg [APR: 1.20, 95% CI: 1.03-1.39; p=0.015] were more likely to be formula-fed compared to their counterparts.

Conclusion

Formula feeding was observed in over half of the infants in our study. Targeted interventions are essential to reduce formula-feeding practices, especially among low-birth-weight infants and those born in private hospitals.

## Introduction

Infant formula is widely used as a processed food alternative for infants under one year of age and is available in liquid and powder forms. Developing countries, including India, exhibit a diverse range of infant feeding practices that include formula feeding [1-4]. Asia accounts for approximately 68.42% of the global infant formula market, with Southeast Asia representing a significant fraction relative to its population [5]. This surge encompasses not only infant formula (from zero to six months) but also follow-up (seven to 12 months) and toddler (13-36 months) formulas, which can negatively impact breastfeeding practices if marketed and consumed inappropriately [6]. A report by the World Health Organization (WHO) and the United Nations Children’s Fund (UNICEF) revealed that more than half of the parents and pregnant women had been targeted by formula food marketing, often in violation of international standards [7].

Breastfeeding practices worldwide are influenced by various factors, including education, psychosocial barriers, and cultural beliefs [8]. A study across 16 sub-Saharan African countries found that mothers with primary education were more likely to follow recommended breastfeeding practices. In contrast, those with higher education showed no significant difference compared to uneducated mothers [9]. This suggests that basic education may improve awareness, but other factors, such as work pressures, may offset the benefits of advanced education. Psychosocial barriers such as stress, shame, nipple pain, societal stigma, lack of family support, and unsupportive social environments hinder breastfeeding and discourage mothers from continuing it. [10-12]. Cultural beliefs further impact practices. In China, some mothers believe that it is better not to start breastfeeding than to stop due to weaning, leading them to rely on infant formula or alternative feeding methods [13]. Addressing these barriers requires targeted interventions, including education, support systems, and breastfeeding-friendly environments. Although the literature on formula feeding and associated factors is available from several countries, there is a substantial gap in the research examining formula-feeding practices and their associated factors in the Indian context [14].

Societal norms, economic pressures, and traditional beliefs about infant nutrition often interact with modern marketing and the accessibility of formula products [15]. Addressing this gap is crucial for tailoring health initiatives to support informed choices, promote optimal infant nutrition, and mitigate adverse impacts on infant health and development. Understanding the formula-feeding practices specific to India is essential for creating targeted interventions that ensure positive child health outcomes and growth. To address this need, we conducted a study to determine the prevalence and factors associated with formula-feeding practices among mothers of infants aged less than one year in a sub-district of Kerala, India.

## Materials and methods

Study design and study setting

This community-based cross-sectional analytical study was conducted among mothers of infants aged 0-12 months residing in a sub-district of Kerala.

Study duration

The study duration was six months, with data collected between February and April 2024.

Sample size estimation

We used non-exclusive breastfeeding as a proxy to calculate the sample size for determining the prevalence of formula feeding. Assuming a prevalence of 44.5% [15], with a 7% absolute precision, a 95% confidence level, and a design effect of 1.5 to adjust for cluster sampling, the estimated minimum sample size was 290 [16]. This was rounded off to 300 to facilitate the recruitment of 10 mothers from each of the 30 clusters.

Sampling technique

**Figure 1:**
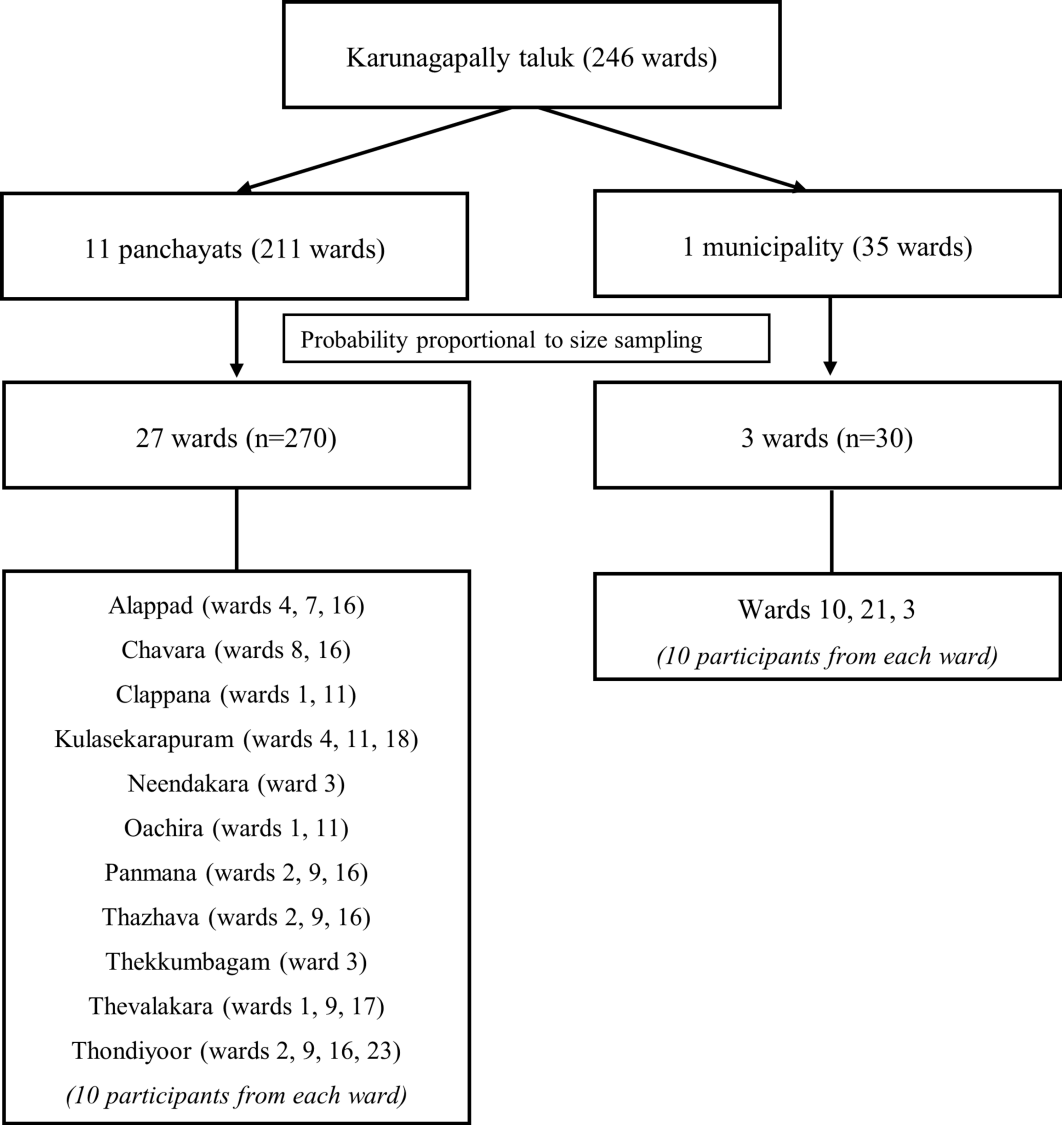
Sample selection process

Cluster sampling was employed to select the mothers of infants for the study. Karunagapally Taluk (sub-district), comprising one municipality (urban area) with 35 wards and 11 panchayats (rural areas) with 211 wards, was chosen as the study area. Each ward, the smallest administrative unit in the local government in Kerala, was considered a cluster. Twenty-seven wards from the rural area and three wards from the urban area were selected based on the population proportional to size. The total population of 428,802 in the taluk was divided by the number of clusters to calculate a sampling interval of 14,293. Starting with a random number (6130), wards were sequentially selected at intervals of 14,293 until 30 wards (clusters) were identified (Figure 1).

In each selected ward, the first household was determined by locating the center of the ward with the help of a local guide. The WHO cluster sampling technique was applied to identify the first household and subsequent households within each ward until 10 mothers were recruited for the study [17].

Data collection

The structured interview schedule used in the study was adapted from internationally validated tools, including the UNICEF/WHO breastfeeding questionnaire [18] and the Centers for Disease Control and Prevention (CDC) guidelines on breastfeeding and infant feeding practices [19]. This adaptation ensured strong content validity by covering critical aspects of breastfeeding practices and infant feeding strategies as recognized in global health research. To further enhance validity, the questionnaire was reviewed by three experts in maternal and child health, epidemiology, and public health, ensuring cultural appropriateness and alignment with the study’s objectives. Reliability was established through a pilot study conducted among a subset of participants with characteristics similar to those of the study population. This pilot helped identify ambiguities and inconsistencies, allowing necessary modifications to improve clarity of the questions and ensure consistent responses.

Study variables 

The independent variables in this study included the mother’s age, categorized as ≤27 years and >27 years; the mother’s educational status, classified into graduates/postgraduates and higher secondary or below; and the mother’s occupation, divided into homemaker and others. The spouse’s occupation was categorized as government or private employee, while socio-economic status was classified based on India’s system for providing ration cards as above poverty line (APL) or below poverty line (BPL) category. Other variables included the type of delivery, recorded as lower segment cesarean section (LSCS) or vaginal delivery, and the place of delivery was categorized as private or government hospital. Antenatal and perinatal factors such as birth weight (<2.5 kg or ≥2.5 kg) and gestational age (<37 weeks or ≥37 weeks) were also measured. Initiation of breastfeeding was recorded as ≤1 hour or >1 hour after birth, and the current age of the infant was categorized as ≤6 months or >6 months. The dependent variable was the practice of formula feeding, defined as any instance of formula feeding during infancy, with infants receiving formula milk at any time classified as formula-fed. 

Ethical consideration

The study protocol was approved by the Institutional Ethics Committee of Amrita School of Medicine, Amrita Institute of Medical Sciences, Kochi (Reference number: ECASM-AIMS-2024-062). Written informed consent was obtained from all participants prior to the commencement of the study.

Data analysis

Data were entered into the EpiCollect 5 mobile application (Centre for Genomic Pathogen Surveillance, Cambridgeshire, UK) based at the Wellcome Sanger Institute) and analyzed using Stata software version 14 (StataCorp LLC, College Station, TX, US). Categorical data were presented as percentages, while continuous data were summarized as mean and standard deviation. The prevalence of formula-feeding practices was calculated with 95% confidence intervals (CIs). Associations between various factors and formula-feeding practices were assessed using the Chi-square test. Unadjusted prevalence ratios (UPRs) and 95% CIs were estimated. Variables with a p value of <0.2 in the bivariate analysis were included in a log-binomial regression analysis to calculate adjusted prevalence ratios (APRs) with 95% CIs. A p value of <0.05 was considered statistically significant.

## Results

The mean (SD) age of the mothers was 27.06 (3.90) years. Among them, 181 (60.33%) were aged ≤27 years and 225 (75%) were graduates or postgraduates. The majority of them (n=202, 67.33%) were homemakers, and 174 (58%) belonged to the APL category. Early initiation of breastfeeding (≤1 hour after birth) was reported by 195 (65%) participants, and the majority of the infants (n=203, 68%) were aged less than six months (Table 1).

**Table 1:** Frequency distribution of the socio-demographic, antenatal, perinatal, and postnatal characteristics of study participants from Karunagapally Taluk, Kollam district (n=300) LSCS: Lower segment cesarean section, kg: kilograms, APL: above poverty line, BPL: below poverty line. APL and BPL were used as per India's socio-economic classification system.

Variables	n	%
Age in years		
≤27	181	60.33
>27	119	39.67
Educational status		
Graduates/postgraduates	225	75.00
Higher secondary and below	75	25.00
Occupation		
Homemaker	202	67.33
Others	98	32.67
Occupation of the husband		
Government employee	56	18.67
Private employee	244	81.33
Socio-economic status		
APL	174	58.00
BPL	126	42.00
Type of delivery		
LSCS	157	52.33
Vaginal delivery	143	47.67
Place of delivery		
Private hospital	160	53.33
Government hospital	140	46.67
Initiation of breastfeeding		
≤1 hour after birth	195	65.00
> 1 hour after birth	105	35.00
Birth weight		
<2.5kg	32	10.67
≥2.5kg	268	89.33
Gestational age in weeks		
<37	122	40.67
≥37	178	59.33
Current age of the child (months)		
≤6	203	67.67
>6	97	32.33

The prevalence of formula-feeding practices reported by the mothers of infants under one year of age was 53.3% (95% CI: 47.5-59.1%), with 63.3% of them from urban areas and 52.2% from rural areas. Table 2 presents the factors associated with formula feeding. Mothers who delivered in private hospitals were significantly more likely to report formula feeding (APR: 3.44, 95% CI: 2.47-4.79; p<0.001) compared to those who delivered in public facilities. Additionally, infants with a birth weight of less than 2.5 kg were more likely to be formula-fed (APR: 1.20, 95% CI: 1.03-1.39; p=0.015).

**Table 2:** Results of bivariate and multivariable regression analysis of the socio-demographic factors and other variables (perinatal, postnatal, and infant health) associated with the formula-feeding practices (n=300) LSCS: lower segment cesarean section, UPR: unadjusted prevalence ratio, APR: adjusted prevalence ratio, CI: confidence interval, kg: kilograms. Variables with a p-value of <0.2 in the unadjusted analysis were included in the adjusted analysis and a p-value of <0.05 was considered statistically significant.

Variables	n	Formula feeding		UPR (95%CI)	APR (95% CI)	P value
		Yes [n (%)]	No [n (%)]			
Mother's education						
Graduation and above	225	131 (58.22)	94 (41.78)	1.50 (1.10-2.04)	1.06 (0.84-1.34)	0.608
Up to higher secondary	75	29 (38.67)	46 (61.33)	1	1	-
Occupation of the husband						
Government employee	56	35(62.50)	21 (37.50)	1.22 (0.96-1.55)	1.14 (0.93-1.41)	0.211
Private employee	244	125 (51.2)	119 (48.8)	1	1	-
Type of delivery						
LSCS	143	85 (59.44)	58 (40.56)	1.24 (1.0-1.53)	1.17 (0.98-1.40)	0.082
Vaginal delivery	157	75 (47.77)	82 (52.23)	1	1	-
Place of delivery						
Private hospital	160	129 (80.62)	31(19.38)	3.64 (2.64-5.01)	3.44 (2.47-4.79)	<0.001
Government hospital	140	31 (22.14)	109 (77.86)	1	1	-
Birth weight						
<2.5kg	32	29 (90.62)	3 (9.38)	1.85 (1.57-2.18)	1.20 (1.03-1.39)	0.015
≥2.5kg	268	131 (48.88)	137 (51.12)	1	1	-
Gestational age						
<37 weeks	122	71 (58.20)	51 (41.80)	1.16 (0.94-1.43)	1.12 (0.93-1.33)	0.225
≥37 weeks	178	89 (50.00)	89 (50.0)	1	1	-
Current age of the child (months)						
≤6	203	110 (54.19)	93 (45.81)	1.05 (0.83-1.32)	1.07 (0.89-1.30)	0.457
>6	97	50 (51.55)	47 (48.45)	1	1	-

## Discussion

The current study found that 53.3% of mothers reported they formula-fed their infants, indicating that formula feeding is widely practiced during infancy. This finding aligns with statistics from the National Family Health Survey (NFHS)-5, which reported non-exclusive breastfeeding rates of 55.5% in Kerala [15]. In this study, the prevalence of formula feeding among mothers of infants under one year was higher in the urban areas (63.3%) compared to rural areas (52.2%), consistent with the pattern observed in exclusive breastfeeding rates across Kerala, Tamil Nadu, and Karnataka in NFHS-5 data. Kerala showed slightly higher exclusive breastfeeding rates in rural (56.0%) than urban (55.0%) areas, while Tamil Nadu and Karnataka also reported minimal urban-rural differences. This suggests a regional trend where urban areas may adopt formula-feeding practices more frequently, potentially influenced by lifestyle and socio-economic factors [15]. Aggressive marketing strategies employed by the infant formula companies significantly influence parental decisions on infant feeding [7]. These marketing practices often undermine breastfeeding by promoting formula feeding as a convenient or superior alternative. The prevalence rates observed in various studies, including the current one, underscore the impact of such market dynamics.

Higher formula-feeding rates have been reported in regions like East Malaysia (73.7%) and China (88%), likely due to a stronger market presence and socio-economic factors that make formula feeding more appealing or accessible [20-22]. In contrast, countries such as Poland (42%) and Egypt (47%) report lower rates, reflecting the influence of cultural values, economic conditions, and regulatory controls on formula marketing [3,23,24]. These variations highlight the critical role of local cultural norms, socio-economic status, and policies in shaping feeding practices. In regions with a robust formula market, factors such as extensive marketing, product availability, and socio-economic conditions favoring formula use contribute to its widespread acceptance. To mitigate the impact of commercial influences, stricter enforcement of the International Code of Marketing of Breast-Milk Substitutes is essential. Public awareness campaigns can educate parents on the long-term health benefits of exclusive breastfeeding, countering the effects of formula marketing. Additionally, practical support systems such as community-based programs, workplace accommodations, and trained healthcare professionals are necessary to foster a sustainable breastfeeding culture.

This study also found that mothers who delivered in private hospitals were more likely to report formula feeding their infants compared to those who delivered in public healthcare facilities. This association between the place of delivery and infant feeding practices aligns with previous research by Odom et al. (2013), highlighting the influence of healthcare environments on feeding patterns through factors such as availability of lactation support, hospital policies, and perceived quality of care [25]. In private hospitals, cultural norms and breastfeeding support systems may differ, leading to fewer breastfeeding-friendly policies and guidance [26]. These findings underscore the need for hospital practices that actively support breastfeeding, as access to environments prioritizing lactation support and baby-friendly policies play a crucial role in shaping mothers' feeding decisions [27].

Furthermore, this study observed that low birth weight infants (<2.5 kg) were more likely to be formula-fed than those with a birth weight of 2.5 kg or above. This trend is consistent with existing research, which indicates that health status factors such as birth weight and medical complications significantly affect feeding patterns [28]. Mothers of low-birth-weight infants often turn to formula feeding due to concerns about their infant’s growth, development, and nutritional needs, perceiving breastfeeding alone as insufficient [29]. Additional barriers for this group include breastfeeding difficulties such as low energy levels or weaker sucking reflexes in the infant, and logistical challenges like mother-infant separation for medical interventions. Such separation can hinder the establishment of breastfeeding, further increasing reliance on formula feeding [30]. In our study, babies born by the LSCS were more likely to be formula fed compared to those born by vaginal deliveries. However, this finding was significant only in the bivariate analysis and did not reach statistical significance in the adjusted analysis. In many cases, specialized feeding approaches, including formula supplementation, are necessary to meet the unique health needs of low-birth-weight infants, contributing to the observed feeding practices in this population.

Strengths and limitations

To the best of our knowledge, this is the first study from India to assess the prevalence and factors associated with formula-feeding practices. However, it has a few limitations. The cross-sectional design restricts the ability to establish causal relationships between the identified factors and formula-feeding practices. Additionally, as the study was conducted in a single taluk, the findings may not be generalizable to other regions. Furthermore, recall bias and social desirability bias could have impacted the accuracy of self-reported feeding practices, with mothers potentially underreporting formula feeding.

## Conclusions

More than half of the mothers reported using infant formula to feed their children. Infants born in private hospitals and low-birth-weight infants were significantly more likely to be formula-fed compared to their counterparts. Targeted interventions are essential to reduce formula feeding, particularly among low-birth-weight infants and those born in private hospitals. Strengthening breastfeeding support systems and educating mothers on the benefits of exclusive breastfeeding and the risks associated with formula feeding are critical steps toward promoting optimal infant nutrition and health outcomes.

## References

[1] Bakshi S, Paswan VK, Yadav SP (2023). A comprehensive review on infant formula: nutritional and functional constituents, recent trends in processing and its impact on infants' gut microbiota. Front Nutr.

[2] (2024). Infant Formula Preparation and Storage. https://www.cdc.gov/nutrition/infantandtoddlernutrition/formula-feeding/infant-formula-preparation-and-storage.html.

[3] Taye AA, Asegidew W, Taderegew MM, Bizuwork YG, Zegeye B (2021). Formula feeding practice and associated factors among mothers with infants 0-6 months of age in Addis Ababa, Ethiopia: a community-based cross-sectional study. Ital J Pediatr.

[4] Martin CR, Ling PR, Blackburn GL (2016). Review of infant feeding: key features of breast milk and infant formula. Nutrients.

[5] (2024). Infant Formula Market. Pharmacy/ Medical Stores, Specialty Stores, and Others), and Regional Forecast, 2024-2032 [Internet.

[6] Baker P, Smith J, Salmon L (2016). Global trends and patterns of commercial milk-based formula sales: is an unprecedented infant and young child feeding transition underway?. Public Health Nutr.

[7] (2024). More than half of parents and pregnant women exposed to aggressive formula milk marketing - WHO/UNICEF. https://www.who.int/news/item/22-02-2022-more-than-half-of-parents-and-pregnant-women-exposed-to-aggressive-formula-milk-marketing-who-unicef.

[8] Rollins NC, Bhandari N, Hajeebhoy N (2016). Why invest, and what it will take to improve breastfeeding practices?. Lancet.

[9] Wako WG, Wayessa Z, Fikrie A (2022). Effects of maternal education on early initiation and exclusive breastfeeding practices in sub-Saharan Africa: a secondary analysis of demographic and health surveys from 2015 to 2019. BMJ Open.

[10] Roe B, Whittington LA, Fein SB, Teisl MF (1999). Is there competition between breast-feeding and maternal employment?. Demography.

[11] Vázquez-Osorio IM, Vega-Sánchez R, Maas-Mendoza E, Heller Rouassant S, Flores-Quijano ME (2022). Exclusive breastfeeding and factors influencing its abandonment during the 1st month postpartum among women from semi-rural communities in southeast Mexico. Front Pediatr.

[12] Murphy R, Foley C, Verling AM, O'Carroll T, Flynn R, Rohde D (2022). Women's experiences of initiating feeding shortly after birth in Ireland: a secondary analysis of quantitative and qualitative data from the National Maternity Experience Survey. Midwifery.

[13] Fei Y, Zhang ZY, Fu WN, Wang L, Mao J (2022). Why do first-time mothers not intend to breastfeed? - A qualitative exploratory study on the decision-making of non-initiation in Jingzhou, China. BMC Pregnancy Childbirth.

[14] Kesavelu D, Dhanasekhar S, Akram W, Rachel A, Sugumaran LB (2024). Optimization of infant nutrition: exploring feeding practices among Indian mothers. Cureus.

[15] (2024). National Family Health Survey (NFHS-5). Mumbai: IIPS.

[16] Ahmed SK (2024). How to choose a sampling technique and determine sample size for research: a simplified guide for researchers. Oral Oncol Rep.

[17] Bennett S, Woods T, Liyanage WM, Smith DL (1991). A simplified general method for cluster-sample surveys of health in developing countries. World Health Stat Q.

[18] UNICEF/WHO UNICEF/WHO (2024). UNICEF/WHO Questionnaire for breastfeeding mother. UNICEF/WHO.

[19] (2024). About Breastfeeding. https://www.cdc.gov/breastfeeding/php/about/index.html.

[20] Abebe L, Aman M, Asfaw S, Gebreyesus H, Teweldemedhin M, Mamo A (2019). Formula-feeding practice and associated factors among urban and rural mothers with infants 0-6 months of age: a comparative study in Jimma zone Western Ethiopia. BMC Pediatr.

[21] Yee CF, Chin R (2007). Parental perception and attitudes on infant feeding practices and baby milk formula in East Malaysia. Int J Consum Stud.

[22] Tang L, Binns CW, Lee AH (2015). Infant formula crisis in China: a cohort study in Sichuan province. J Health Popul Nutr.

[23] Rozensztrauch A, Klaniewska M, Berghausen-Mazur M (2022). Factors affecting the mother's choice of infant feeding method in Poland: a cross-sectional preliminary study in Poland. Ir J Med Sci.

[24] Tawfik S, Saied D, Mostafa O, Salem M, Habib E (2019). Formula feeding and associated factors among a group of Egyptian mothers. Open Access Maced J Med Sci.

[25] Odom EC, Li R, Scanlon KS, Perrine CG, Grummer-Strawn L (2013). Reasons for earlier than desired cessation of breastfeeding. Pediatrics.

[26] Bengough T, Dawson S, Cheng HL (2022). Factors that influence women's engagement with breastfeeding support: a qualitative evidence synthesis. Matern Child Nutr.

[27] Flacking R, Ewald U, Nyqvist KH, Starrin B (2006). Trustful bonds: a key to "becoming a mother" and to reciprocal breastfeeding. Stories of mothers of very preterm infants at a neonatal unit. Soc Sci Med.

[28] Brown A, Jordan S (2013). Impact of birth complications on breastfeeding duration: an internet survey. J Adv Nurs.

[29] Li R, Perrine CG, Anstey EH, Chen J, MacGowan CA, Elam-Evans LD (2019). Breastfeeding trends by race/ethnicity among US children born from 2009 to 2015. JAMA Pediatr.

[30] Victora CG, Bahl R, Barros AJD (2016). Breastfeeding in the 21st century: epidemiology, mechanisms, and lifelong effect. Lancet.

